# Supplementary material for: Oceanic mesoscale eddies as crucial drivers of global marine heatwaves
Source: Nat Commun. 2023 May 23;14:2970. doi: 10.1038/s41467-023-38811-z (PMC10206097; doi:10.1038/s41467-023-38811-z)
Supplement: Supplementary file 1 — Supplementary Information [file 41467_2023_38811_MOESM1_ESM.pdf]

Supplementary Information

Oceanic Mesoscale Eddies as Crucial Drivers of Global Marine Heatwaves

Ce Bian[1,2], Zhao Jing[1,2], Hong Wang[1,2], Lixin Wu[1,2], Zhaohui Chen[1,2], Bolan Gan[1,2] and Haiyuan Yang[1,2]

[1]Frontiers Science Center for Deep Ocean Multispheres and Earth System and Key Laboratory of Physical Oceanography, Ocean University of China, Qingdao, China.

[2]Laoshan Laboratory, Qingdao, China

Corresponding author: Zhao Jing (jingzhao@ouc.edu.cn)

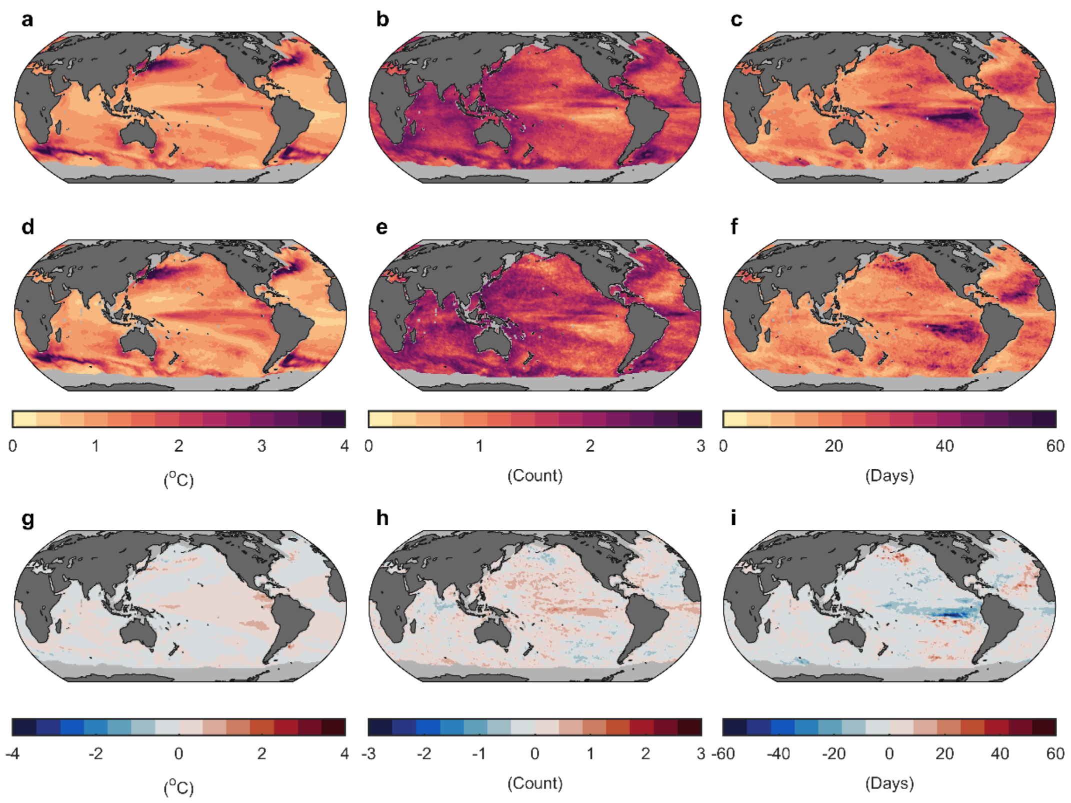

Supplementary Figure 1| Marine heatwave (MHW) statistics derived based on different temperature indices and baseline periods. Climatological mean values of MHW intensity **(a)**, frequency **(b)**, and duration **(c)** derived based on the CESM-H SST during 1982-2021. **d-f,** are the same as **a-c,** but based on the vertical mean temperature in the upper 50 m during 1920-1934. **g-i** are the difference between **d-f** and **a-c**.

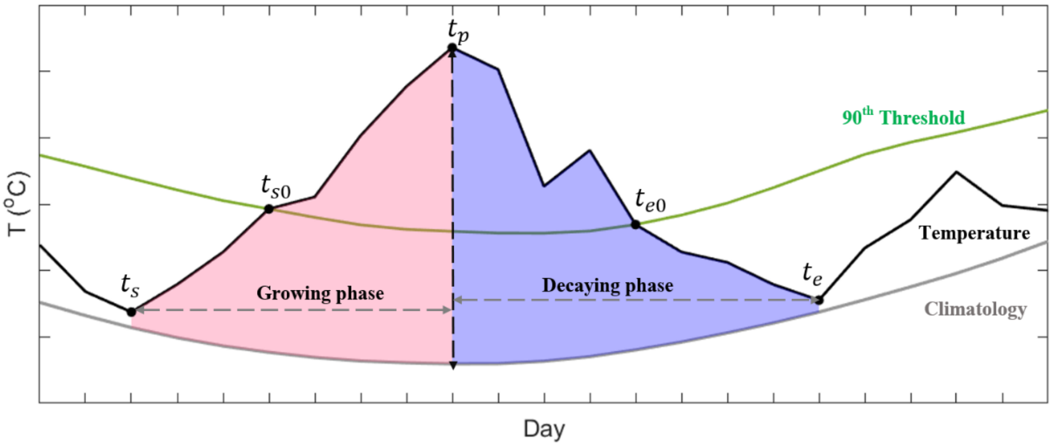

Supplementary Figure 2| Schematic of an marine heatwave (MHW) life cycle. The black line shows an example of SST time series. The grey and green lines show the climatological mean SST seasonal cycle and seasonally varying SST threshold (i.e., the 90th percentile) during the baseline period 1982-2021 computed following Hobday et al.[1], respectively. The t_s and t_e correspond to the start and end times of the MHW defined in this study, whereas t_{s0} and t_{e0} correspond to those defined by Hobday et al.[1]. The t_p corresponds to the peaking time of the MHW separating the growing (red shading) and decaying phases (blue shading), respectively.

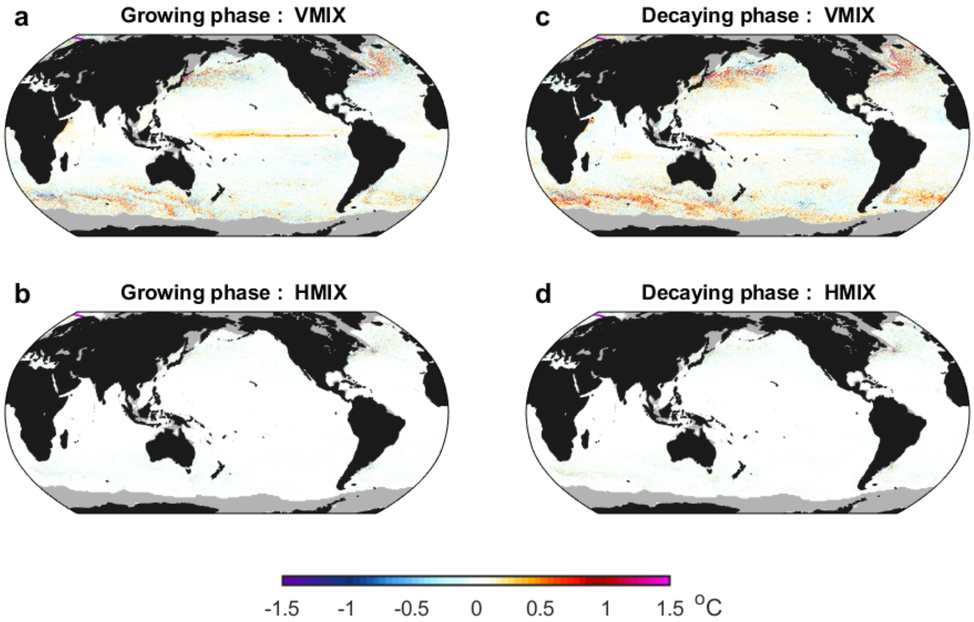

Supplementary Figure 3| Decomposition of subgrid-scale mixing (MIX) into vertical mixing and horizontal mixing components. Contribution to the $\langle T_a \rangle$ change during the growing phase of marine heatwaves (MHWs) averaged at each grid point by the subgrid-scale vertical mixing (VMIX) **(a)** and horizontal mixing (HMIX) **(b)**. **c,d,** are the same as **a,b,** but for the decaying phase of the MHWs. Grids with temporary or permanent sea-ice coverage in the observation are masked by grey.

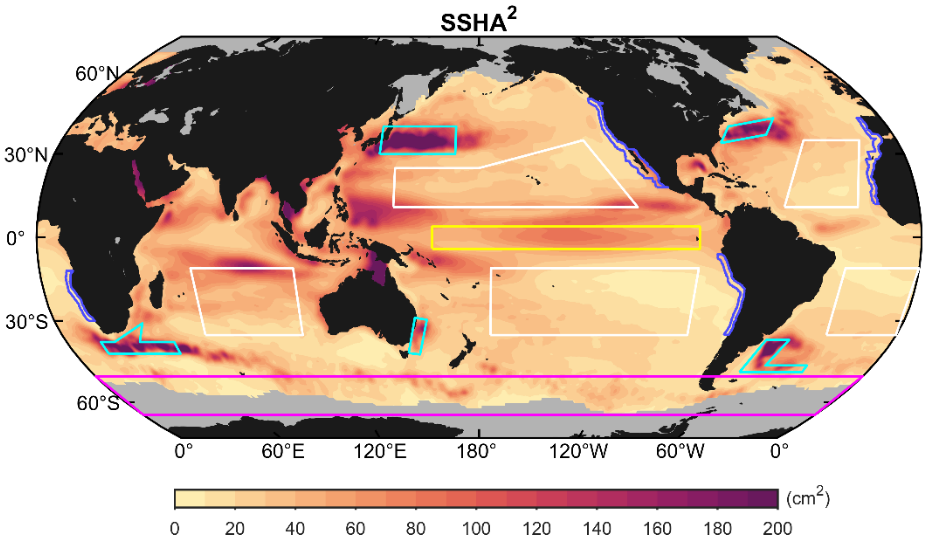

Supplementary Figure 4| Domains of different regions. The western boundary currents and their extensions (encompassed by light blue line), the Southern Ocean (pink lines), the central-to-eastern equatorial Pacific (yellow lines), the eastern boundary upwelling systems (deep blue lines), and the subtropical gyre interior (white lines). Shading represents the variance of mesoscale sea surface height anomaly during 1993-2021 derived from the satellite altimeters.

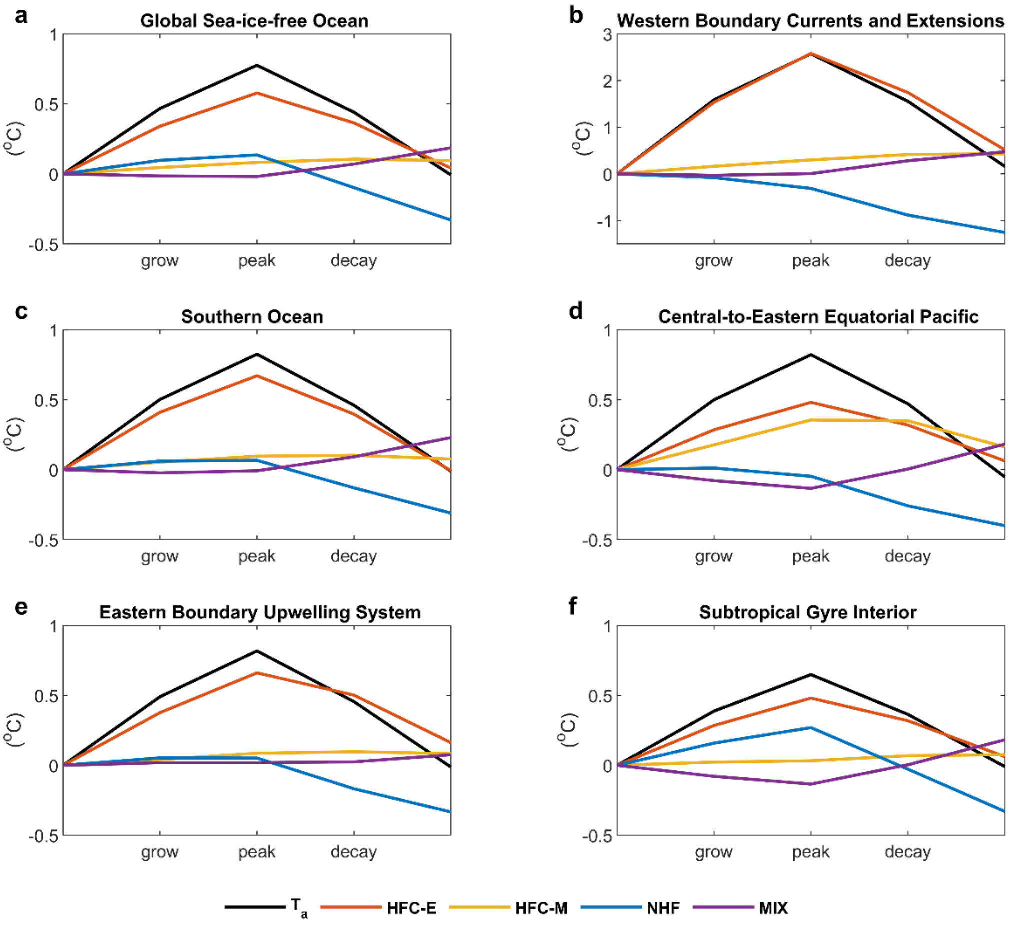

Supplementary Figure 5 | Same as Figure 3 but for the vertical average over the upper 20 m.

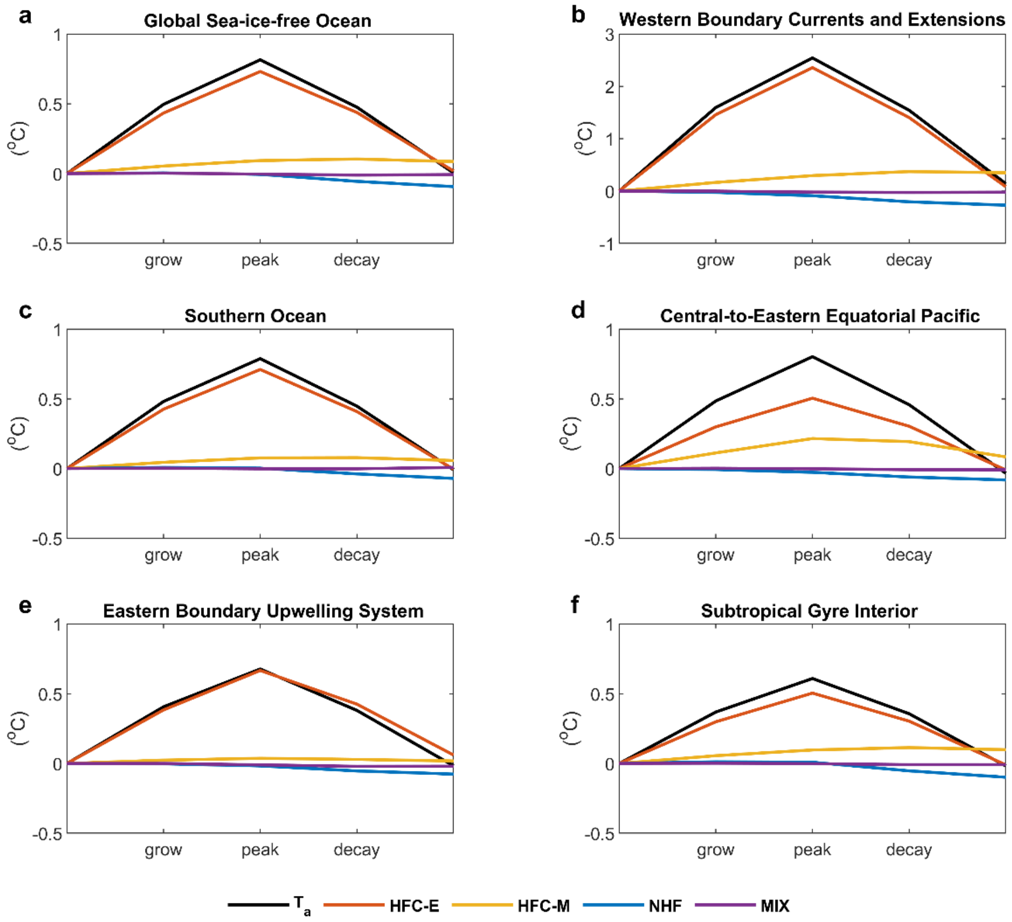

Supplementary Figure 6 | Same as Figure 3 but for the vertical average over the upper 100 m.

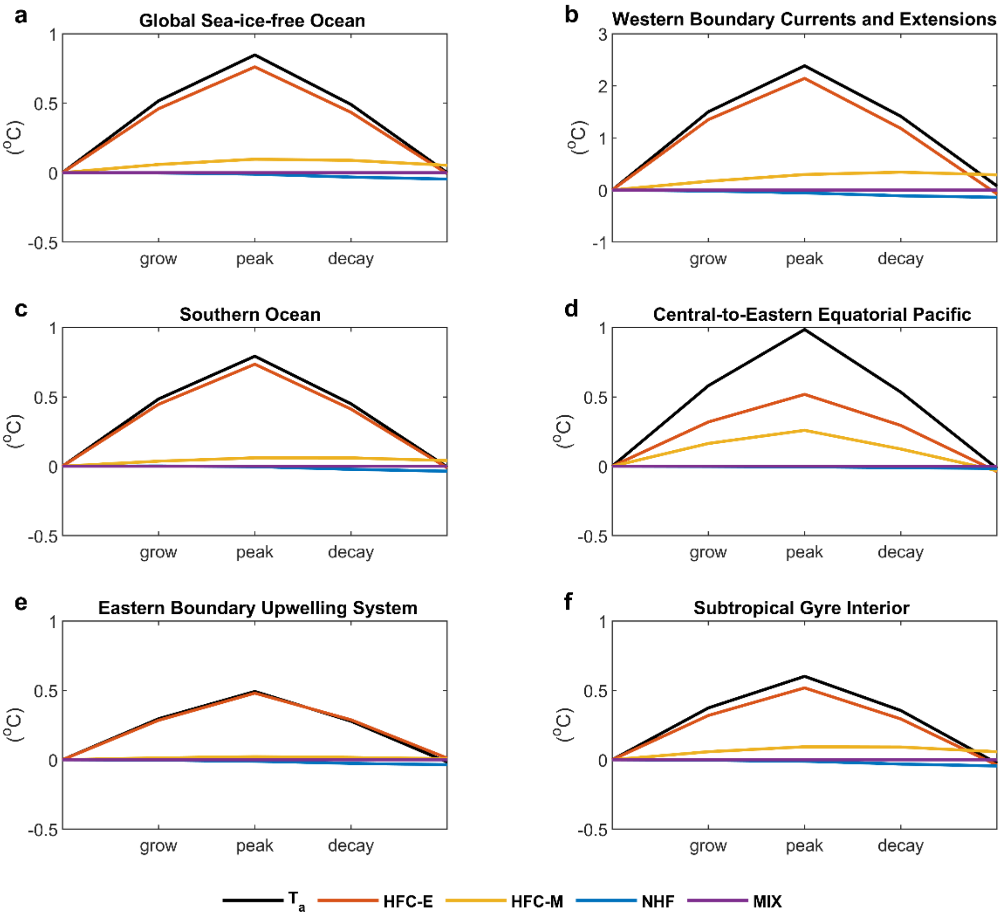

Supplementary Figure 7 | Same as Figure 3 but for the vertical average over the upper 200 m.

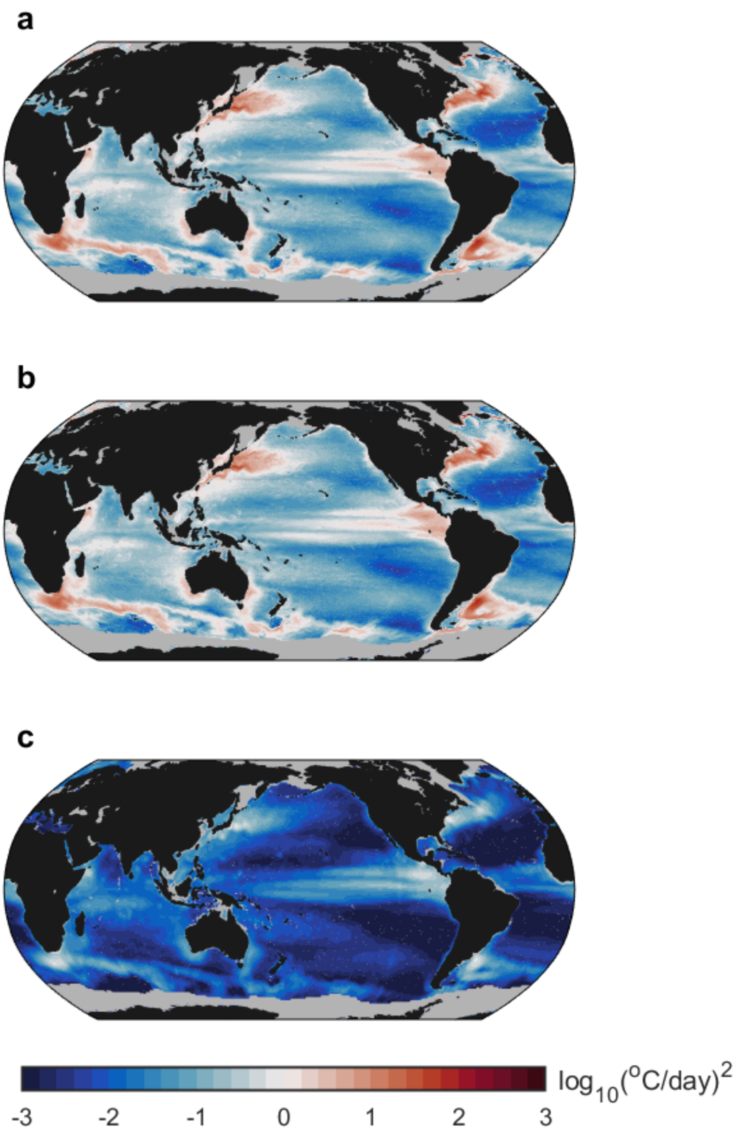

Supplementary Figure 8| Decomposition of heat flux convergence by mesoscale eddies (HFC-E) into the mesoscale and large-scale components. Variance of HFC-E **(a)**, mesoscale HFC-E **(b)**, and large-scale HFC-E **(c)** averaged over the upper 50 m during 1920-1934 simulated by the CESM-H.

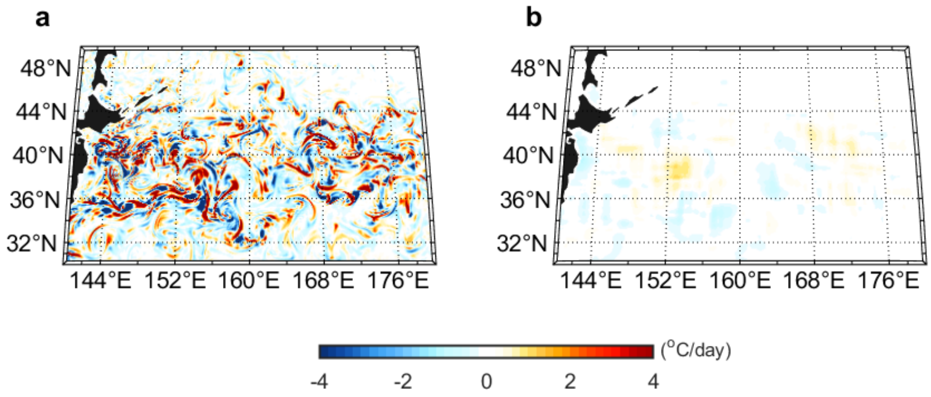

Supplementary Figure 9 | Snapshots of the (a) HFC-E and (b) spatially low-pass filtered HFC-E in the Kuroshio extension on May 16th 1924.

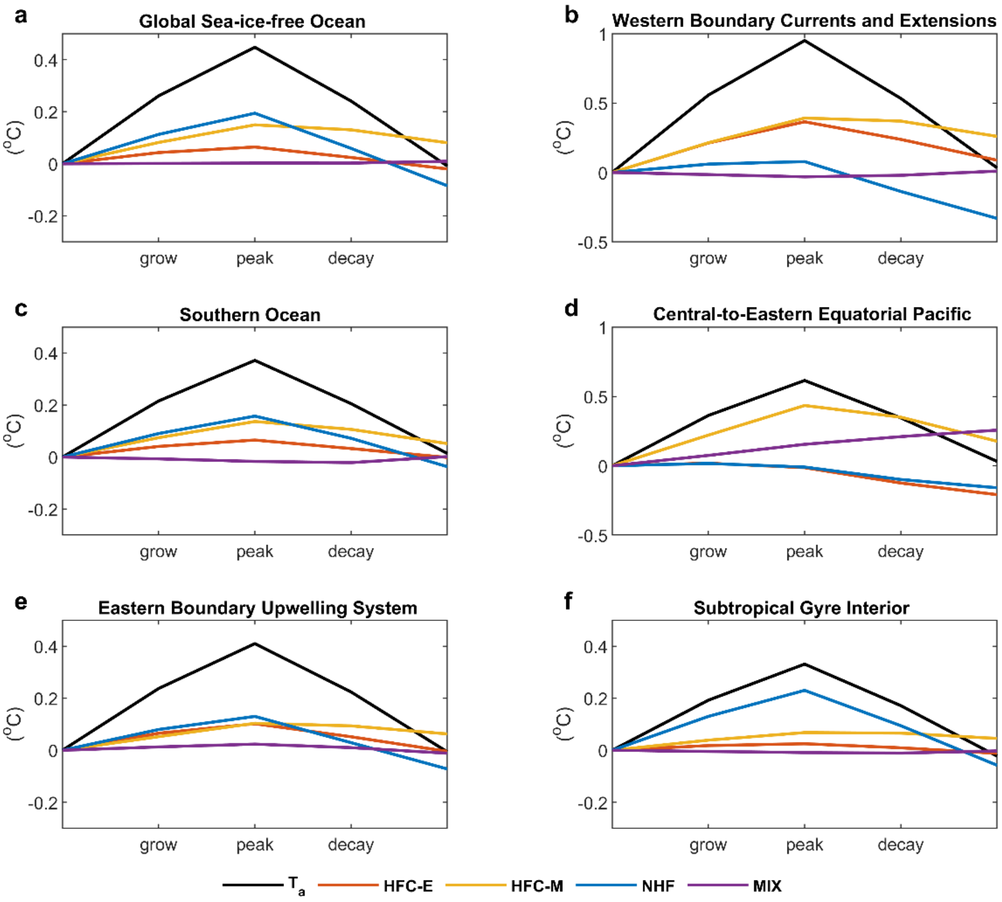

Supplementary Figure 10 | Same as Figure 3 but for the large-scale MHWs.

Supplementary Table 1 | A list of coarse-resolution CGCMs in CMIP6 used in this study. The CGCMs here are the same as those used by Wang et al.[2].

CMIP6	Oceanic Resolution	CMIP6	Oceanic Resolution
ACCESS-CM2	1°	HadGEM3-GC31-LL	1°
ACCESS-ESM1-5	1°	HadGEM3-GC31-MM	0.25°
BCC-CSM2-MR	1°	IPSL-CM6A-LR	1°
CESM2-WACCM	1°	MIROC6	1°
CESM2	1°	MPI-ESM1-2-HR	0.7°
CNRM-CM6-1	1°	MPI-ESM1-2-LR	1°
CNRM-ESM2-1	1°	MRI-ESM2-0	1°
EC-Earth3-Veg-LR	1°	NESM3	1°

Supplementary References

1. Hobday, A. J. *et al.* A hierarchical approach to defining marine heatwaves. *Progress in Oceanography* **141**, 227–238 (2016).
2. Wang, S. *et al.* Changing ocean seasonal cycle escalates destructive marine heatwaves in a warming climate. *Environmental Research Letters* **17**, (2022).
